# Bergamot Leaf Extract Is Associated With Reduced Hepatic DNA Strand Breaks in Diet‐Induced Obese Rats

**DOI:** 10.1002/em.70074

**Published:** 2026-08-21

**Authors:** Luan H. S. Moreira, Jordanna Cruzeiro, Taynara A. Vieira, Tony F. Grassi, Mariane A. P. Silva, Maria Vitória Destro, Camila R. Corrêa, Mariana G. Braz

**Affiliations:** ^1^ São Paulo State University (UNESP), Medical School, Experimental Research Unity (UNIPEX) Botucatu Brazil; ^2^ São Paulo State University (UNESP), Medical School, Division of Anesthesiology, GENOTOX Laboratory Botucatu Brazil; ^3^ São Paulo State University (UNESP), Medical School, Department of Pathology, RedOx and Inflammation Laboratory Botucatu Brazil

**Keywords:** *Citrus bergamia*, comet assay, DNA damage, liver, obesity

## Abstract

Obesity is a major global public health challenge associated with chronic low‐grade inflammation and oxidative stress. Although natural antioxidant compounds have been investigated to mitigate obesity‐related molecular damage, the effects of bergamot ( *Citrus bergamia* ) leaf extract (BLE) on DNA integrity remains unclear. This study evaluated the effects of BLE on hepatic DNA damage in diet‐induced obese rats. Forty Wistar rats were fed either a control or a high‐sugar‐fat (HSF) diet and treated with BLE by oral gavage. The adiposity index was recorded for all groups, and hepatic DNA damage was assessed using the comet assay. The HSF diet significantly increased both the adiposity index and DNA strand breaks in the liver compared with the control group. Notably, BLE treatment was associated with a lower adiposity index and reduced hepatic DNA strand breaks measured by the comet assay in obese rats (*p* < 0.05). These findings indicate an association between BLE treatment and reduced hepatic DNA damage in this model of diet‐induced obesity.

## 1 Introduction

Obesity is a complex metabolic disorder characterized by excessive adipose tissue accumulation and is increasingly recognized as a condition associated with systemic oxidative stress and chronic low‐grade inflammation. These alterations contribute to the development of several obesity‐related comorbidities, including Type 2 diabetes, cardiovascular diseases, and certain cancers (Hruby and Hu 2015; Włodarczyk and Nowicka 2019). The prevalence of obesity has risen substantially, making it a major global public health challenge.

One mechanism linking obesity to disease progression involves the excessive production of reactive oxygen species (ROS), leading to an imbalance between oxidant generation and antioxidant defense systems. Elevated ROS levels may damage cellular macromolecules, including lipids, proteins, and nucleic acids, resulting in DNA damage and genomic instability (Dludla et al. 2018). Indeed, a study using the comet assay reported higher frequency of DNA strand breaks in leukocytes from obese individuals, highlighting the association between obesity and genetic damage (Luperini et al. 2015).

Obesity induced by high‐sugar‐fat (HSF) diets is strongly associated with metabolic dysfunction, chronic low‐grade inflammation, and increased oxidative stress, contributing particularly to hepatic injury (Sheikh et al. 2025). A study using this experimental model demonstrated increased systemic and tissue‐specific oxidative stress, including elevated plasma 8‐hydroxy‐2′‐deoxyguanosine levels and increased ROS levels in liver and extrahepatic tissues (Siqueira et al. 2024). However, whether this redox imbalance results in hepatic DNA damage remains unclear.

There is growing scientific interest in natural compounds with antioxidant and anti‐inflammatory properties capable of mitigating the deleterious effects of obesity. Among these compounds, bergamot ( *Citrus bergamia* ), a citrus fruit derived from the hybrid of bitter orange and citron, stands out due to its rich composition of bioactive flavonoids, with antioxidant and lipid metabolism‐modulating properties (Adorisio et al. 2023; Mollace et al. 2011; Vieira et al. 2025). Most available evidence derives from studies using the bergamot polyphenolic fraction obtained from fruit juice, which is rich in flavonoids such as naringin, neohesperidin, neoeriocitrin, brutieridin, and melitidin (Formisano et al. 2019; Moufida and Marzouk 2003). Interestingly, recent analytical investigations suggest that leaves from certain *Citrus* species may have phenolic compositions comparable to or even superior to those of the fruit, with higher polyphenol content and greater antioxidant activity (Baron et al. 2021). In fact, a study with bergamot leaf extract (BLE) has been shown to exert hypolipidemic, anti‐inflammatory, and oxidative stress‐reducing effects in an experimental model of obesity (Nakandakare‐Maia et al. 2023). Additionally, BLE attenuated obesity‐related liver alterations by improving oxidative stress and modulating lipid and fatty acid metabolism (Cruzeiro et al. 2025).

Despite these findings, while the metabolic effects of bergamot are documented, the potential effects of *Citrus bergamia* leaf extract specifically on obesity‐associated hepatic DNA damage remain unexplored. Therefore, the present study aimed to investigate whether treatment with BLE is associated with a reduction in hepatic DNA strand breaks in obese rats, hypothesizing that BLE administration could mitigate obesity‐induced genotoxic stress.

## 2 Materials and Methods

### 2.1 Animals and Experimental Design

The experimental protocol was approved by the Ethics Committee on the Use of Animals of UNESP (#1337/2020). Briefly, 40 male Wistar rats (30 days old) were obtained from the Multidisciplinary Center for Biological Investigation in Laboratory Animal Science (UNICAMP), Brazil, and housed under controlled temperature conditions (24°C ± 2°C) with a 12 h light–dark cycle and free access to food and water.

**FIGURE 1:**
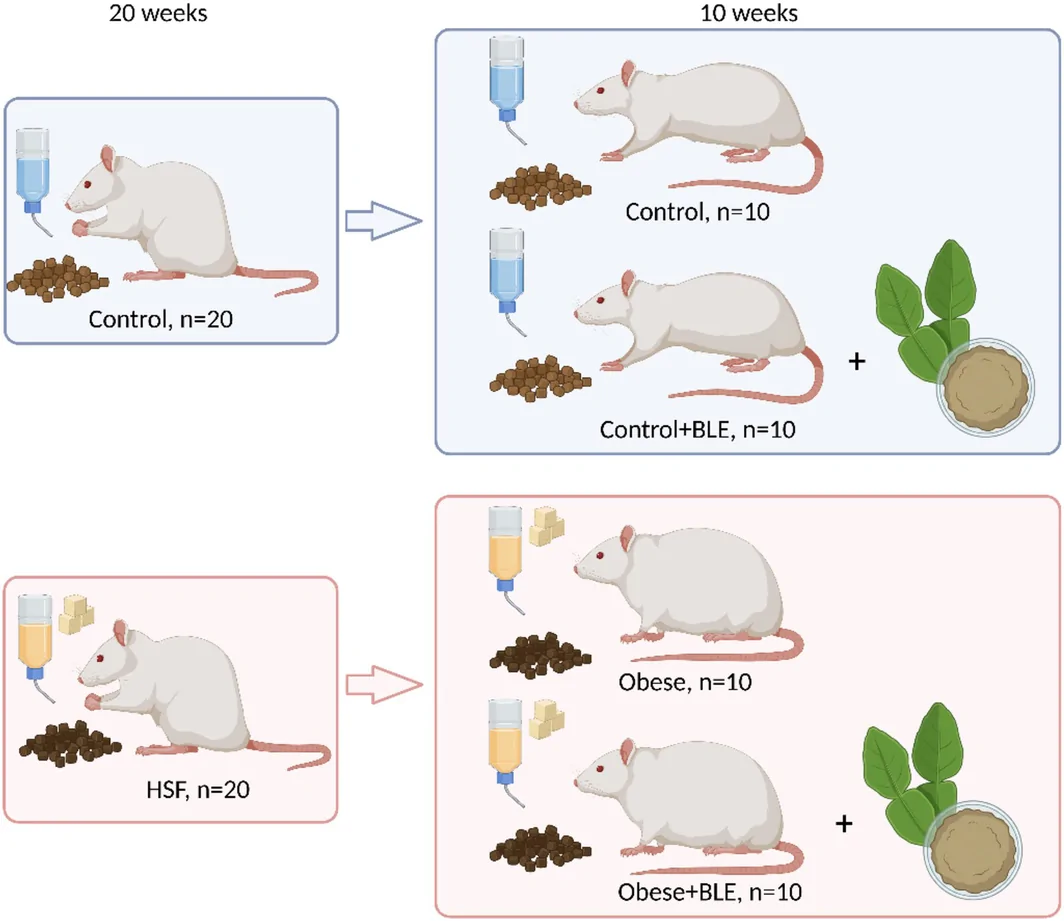
Schematic representation of the experimental protocol. HSF, High sugar‐fat; BLE, *Citrus bergamia* leaf extract. Created with BioRender.com.

After a two‐week adaptation period, the animals were assigned to receive either a standard control diet (C) or an HSF diet supplemented with 25% sucrose in drinking water for 20 weeks to induce obesity. The animals were then reassigned into four experimental groups (*n* = 10/group): Control (C), Control + *Citrus bergamia* leaf extract (C + BLE), Obese (HSF), and Obese + BLE (HSF + BLE). The extract was administered daily by gavage (50 mg/kg body weight) for an additional 10 weeks, while untreated groups received water (Figure 1). Detailed descriptions of the diet composition are available in a previous publication (Francisqueti et al. 2017).

The BLE used in this study originated from a previously characterized batch, and its detailed phytochemical profiling and prior application in experimental obesity models have been documented (Baron et al. 2021; Cruzeiro et al. 2025). Briefly, the chemical composition of BLE comprises specific clusters of limonoids (e.g., nomilin glucoside, nomilinic acid glucoside, limonin), phenolic acids (e.g., feruloyl glucoside and sinapoyl glucoside isomers), flavones (e.g., luteolin, apigenin, chrysoeriol, diosmetin), and flavanonols. The polyphenolic and flavonoid matrix is predominantly characterized by five major flavonoids, quantified as follows: naringin (164.31 mg/g), neohesperidin (144.95 mg/g), neoeriocitrin (133.42 mg/g), brutieridin (59.77 mg/g), and melitidin (31.20 mg/g).

The BLE dose of 50 mg/kg body weight was selected based on prior efficacy and safety data from established experimental models of obesity using the same extract fraction. This specific concentration was recently demonstrated to successfully rescue metabolic, lipid, and oxidative imbalances directly in the liver tissue of obese rats (Cruzeiro et al. 2025). Additionally, this dose has been well‐documented to exert comprehensive multi‐system protection without inducing any systemic toxicity or adverse effects (Siqueira et al. 2022, 2024; Nakandakare‐Maia et al. 2023).

At the end of the protocol, the animals were fasted for 8 h, anesthetized with thiopental (120 mg/kg, intraperitoneally), and euthanized. Liver samples were immediately collected for DNA damage analysis, and adipose tissue was collected to determine the adiposity index. Samples were coded and analyzed in a blinded manner. Experiments were performed in batches including all experimental groups to avoid possible bias.

### 2.2 DNA Damage in Liver

DNA damage was analyzed using the comet assay (Single Cell Gel Electrophoresis—SCGE), following the protocol described by Tice et al. (2000), with modifications (Destro et al. 2025) and in accordance with the Minimum Information for Reporting on the Comet Assay (Møller et al. 2020), under yellow‐light conditions.

Fresh liver samples were collected and processed immediately. The samples were rinsed in ice‐cold phosphate‐buffered saline (PBS) to obtain a homogeneous cell suspension. An aliquot (10 μL) of the suspension was used to assess cell viability by trypan blue exclusion (≥ 70%) using an automated cell counter (Countess Automated Cell Counter, Invitrogen, USA). For slide preparation, 50 μL of the cell suspension was mixed with 150 μL of low‐melting‐point agarose (LMP) and spread onto microscope slides previously precoated with a thin layer of 1.5% normal‐melting‐point agarose (NMP). Coverslips were placed over the slides and removed after solidification.

After solidification, the slides were immersed in freshly prepared, chilled lysis solution (2.5 M NaCl, 100 mM EDTA, 10 mM Tris, pH 10, with 1% Triton X‐100 and 10% DMSO added immediately before use) for 24 h. Subsequently, the slides were washed with PBS and placed in freshly prepared alkaline electrophoresis buffer (1 mM EDTA and 300 mM NaOH, pH > 13) at 4°C for 20 min to allow DNA unwinding. Electrophoresis was conducted at 25 V (0.8 V/cm) and 0.3 A for 20 min. In addition to the negative control (percentage of DNA in the comet tail: 5 ± 2), positive control induced by ethyl methanesulfonate (percentage of DNA in the comet tail: 60 ± 5) was included in each run.

After electrophoresis, the slides were neutralized in 0.4 M Tris buffer (pH 7.5) for 15 min, fixed in absolute ethanol, and allowed to dry at room temperature. Immediately before analysis, the slides were stained with 75 μL of SYBR Green. Nucleoids were visualized under a fluorescence microscope (Olympus BX, Japan) coupled to an image analysis system (Comet Assay IV, Perceptive Instruments, UK) by a single analyzer. A total of 150 nucleoids per animal were analyzed (75 per slide), with two slides per liver. DNA damage was quantified using the tail intensity parameter, expressed as the percentage of DNA in the comet tail.

### 2.3 Statistical Analysis

The Shapiro–Wilk test was used to assess data distribution. Comparisons among the four experimental groups (C, C + BLE, HSF, and HSF + BLE) were performed using two‐way analysis of variance (ANOVA) followed by Tukey's post hoc test for parametric data (DNA damage), which are presented as mean ± standard deviation. For non‐parametric data (adiposity index), the Kruskal‐Wallis test followed by Dunn's post hoc test was applied, and results are expressed as median and interquartile range. To evaluate the relationship between obesity severity and genetic damage at the individual animal level, Spearman's rank correlation coefficient was calculated between the adiposity index and hepatic DNA strands breaks. Furthermore, an Analysis of Covariance (ANCOVA) was performed using the adiposity index as a continuous covariate to determine whether the effect of BLE on hepatic DNA damage was independent of, or mediated by, changes in adipose tissue accumulation. A *p* < 0.05 was considered statistically significant. Statistical analyses were performed using GraphPad Prism software (version 9.0, GraphPad Software, USA) and SPSS Statistics (IBM Corp., USA).

## 3 Results

The HSF group exhibited a higher adiposity index than the control group. In contrast, the HSF + BLE group showed a reduction in the adiposity index compared with the obese group (HSF), although it remained higher than that observed in the control + BLE group (Table 1).

**TABLE 1:** Adiposity index of the experimental groups.

Parameter	Control	Control + BLE	HSF	HSF + BLE
Adiposity index (%)	5.6 (4.7–6.4)	5.6 (5.0–6.6)	9.3 (7.4–11.0)*	7.8 (6.9–9.4) ^#&^

*Note:* Values are presented as median and interquartile range. Differences were considered significant at *p* < 0.05 *vs. Control, ^#^vs. Control + BLE, ^&^vs. HSF.

Abbreviations: BLE, *Citrus bergamia* leaf extract; HSF, high sugar‐fat diet.

**FIGURE 2:**
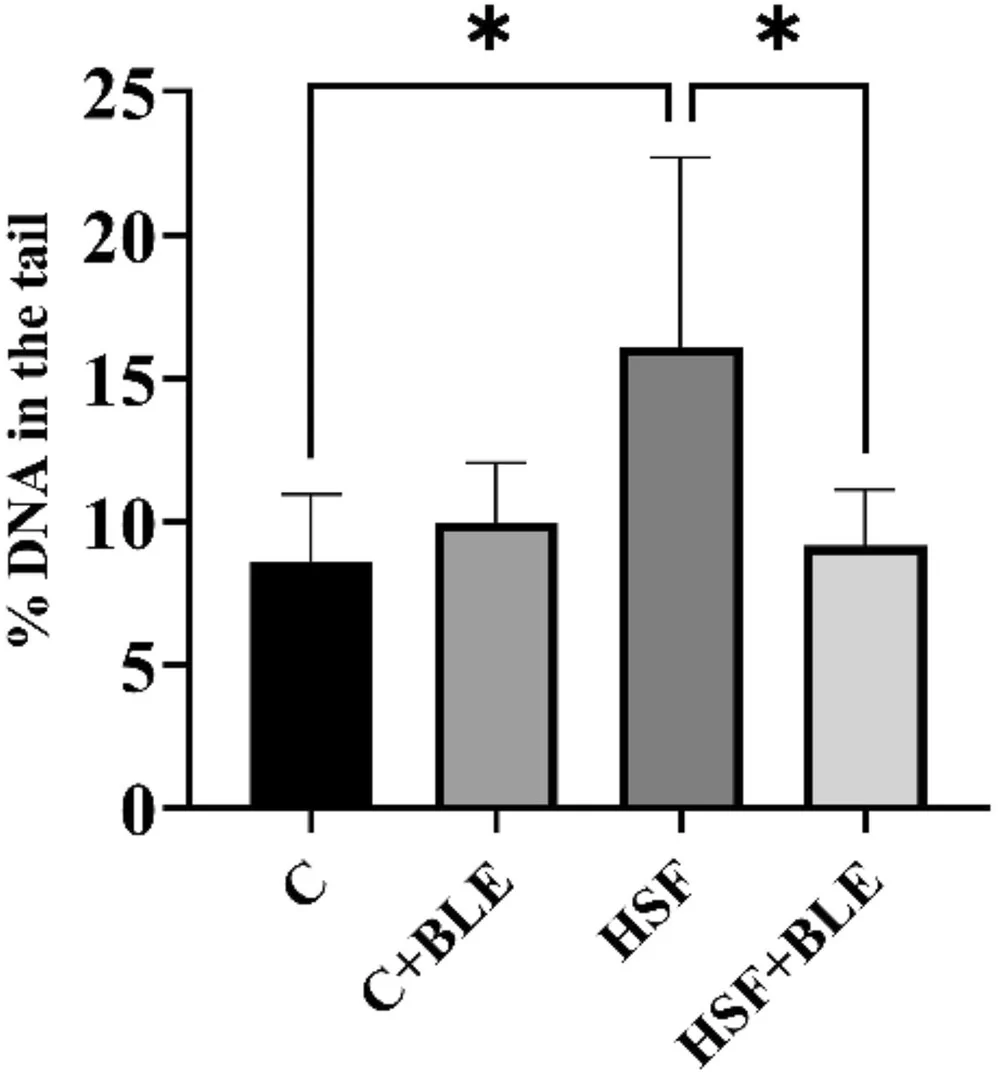
DNA strand breaks evaluated via the comet assay in the liver of the experimental groups. Control (C), Control + *Citrus bergamia* leaf extract (C + BLE), Obese (HSF), and Obese + BLE (HSF + BLE). Data are expressed as mean ± standard deviation. Statistical analysis was performed using one‐way ANOVA followed by Tukey's post hoc test. *Significant differences: HSF versus C (*p* = 0.049) and HSF + BLE versus HSF (*p* = 0.024). No significant differences were observed for C + BLE versus C (*p* = 0.941), HSF + BLE versus C (*p* = 0.994), C + BLE versus HSF (*p* = 0.058), and C + BLE versus HSF + BLE (*p* = 0.975).

Hepatic DNA damage assessed by the comet assay is presented in Figure 2. DNA damage increased approximately twofold in the HSF group compared to control group (*p* < 0.05). There was no significant difference between control + BLE and control groups. Notably, BLE treatment significantly reduced DNA damage in HSF animals (*p* < 0.05), with values approaching those observed in the control group.

To evaluate the interplay between obesity severity and DNA damage at the individual animal level, Spearman's rank correlation was performed. A remarkably strong and highly significant positive correlation was observed between the adiposity index and hepatic DNA strand breaks (*ρ* = 0.83, *p* < 0.0001), mathematically demonstrating that the intensity of hepatic genotoxicity is strictly linked to the severity of fat accumulation. Furthermore, to determine whether the genoprotective effect of BLE was direct or mediated by fat loss, ANCOVA was applied using the adiposity index as a covariate. When statistically adjusting and equalizing the adiposity levels across all groups, the independent effect of BLE on DNA damage became non‐significant (*p* = 0.158). This indicates that the lower hepatic DNA damage observed in the HSF + BLE group is secondary to the robust reduction in adiposity and its associated metabolic stress, rather than a primary, direct tissue‐specific antigenotoxic action.

## 4 Discussion

The present study demonstrated that BLE treatment was associated with reduced hepatic DNA damage in obese rats. While the metabolic effects of bergamot‐derived compounds have been previously reported, the evaluation of hepatic DNA strand breaks in BLE‐treated obese animals represents the specific novel contribution and narrow focus of the present investigation.

The increase in adiposity index reflects excessive energy storage and expansion of white adipose tissue, which is metabolically active and associated with chronic low‐grade inflammation. This condition contributes to adipokine dysregulation and the development of insulin resistance, ultimately impairing glucose homeostasis (Kawai et al. 2021; Samuel and Shulman 2016). These metabolic disturbances are strongly associated with increased ROS production resulting from mitochondrial dysfunction and lipid overload induced by hypercaloric diets (Carresi et al. 2020; Williamson and Sheedy 2020). Obesity has been consistently linked to systemic oxidative stress and metabolic imbalance, which contribute to cellular and molecular damage (Zatterale et al. 2020). In the present study, this effect was observed in the liver, where DNA strand breaks were significantly increased, highlighting the high susceptibility of metabolically active organs to obesity‐induced cellular injury.

Notably, the HSF + BLE group showed a marked reduction in the adiposity index compared with untreated obese animals, successfully restoring fat accumulation to baseline control levels. Previous studies have demonstrated that bergamot‐derived compounds, particularly polyphenols, can modulate lipid metabolism, improve metabolic parameters, and reduce oxidative stress (Mollace et al. 2011; Carresi et al. 2020). Importantly, BLE treatment also significantly reduced hepatic DNA damage in obese animals, demonstrating a protective association against obesity‐associated genetic damage.

In line with these findings, studies have shown that bergamot polyphenols exert protective effects against metabolic disturbances and liver damage in diet‐induced obesity models (Parafati et al. 2015; Adorisio et al. 2023). Cirmi et al. (2016) demonstrated that BLE has a greater protective redox potential and acts more significantly in tissues with high metabolic rates, such as the liver. Crucially, while direct hepatic oxidative stress and inflammatory biomarkers were not evaluated in the present study, the underlying mechanisms of BLE at this exact dose (50 mg/kg) under an identical experimental design have been previously established by our research group. Cruzeiro et al. (2025) demonstrated that BLE‐treated obese rats exhibited a powerful rescue of hepatic oxidative balance, evidenced by a significant downregulation of lipid peroxidation and advanced oxidation protein products, alongside a robust upregulation of superoxide dismutase and catalase activities in the liver tissue. Therefore, the reduction in hepatic DNA strand breaks observed here is highly consistent with the previously demonstrated capacity of BLE to mitigate hepatic oxidative stress and lipid imbalances.

Regarding the experimental conditions, the 8‐h fasting period preceding euthanasia was strictly applied to all experimental groups to standardize metabolic parameters. Because this protocol was identical for all groups, any baseline influence it might have on hepatic oxidative status or DNA damage is unlikely to explain the differences observed between the treated and untreated rats.

To further decipher the interplay between fat accumulation and genotoxicity at the individual animal level, a Spearman's correlation was performed. A remarkably strong and significant positive association was observed between the adiposity index and hepatic genetic damage, confirming that obesity severity strictly drives DNA strand breaks. Furthermore, when adjusting for adiposity as a covariate in an ANCOVA model, the independent effect of BLE on DNA damage became non‐significant. This statistical insight strongly demonstrates that the genoprotective effect of BLE observed in this model is secondary to its capacity to reduce adipose tissue accumulation and its associated systemic metabolic burden, rather than a primary, direct tissue‐specific antigenotoxic mechanism.

This study has some limitations that should be considered when interpreting the results. First, our experimental design included only male rats, a single dose of BLE, and the evaluation of a single tissue (liver) and a single DNA damage endpoint via the standard alkaline comet assay. Furthermore, due to the lack of direct primary causality established by our ANCOVA model, the absence of specific oxidative DNA damage endpoints (such as the Fpg‐modified comet assay or 8‐oxo‐dG measurements) must be acknowledged as limitations.

In conclusion, the findings suggest that HSF diet‐induced obesity promotes metabolic dysfunction associated with increased hepatic DNA damage. Furthermore, BLE treatment was associated with reduced hepatic DNA strand breaks and lower adiposity in obese rats. Rather than reflecting a direct antigenotoxic pathway, this protective effect appears to be secondary to the mitigation of obesity‐related systemic and metabolic stress, supported by prior evidence of hepatic antioxidant rescue exerted by this extract. Future mechanistic and translational studies utilizing multiple doses, female rats, and specific oxidative/inflammatory markers are required to fully characterize the underlying molecular pathways.

## Author Contributions

L.H.S.M., C.R.C., and M.G.B. designed the study. J.C., T.A.V., T.F.G., M.A.P.S., M.V.D., and L.H.S.M. conducted the experiments and analyzed the data. C.R.C. and M.G.B. supervised the study and acquired funding. All authors drafted and approved the final manuscript.

## Funding

This work was supported by Fundação de Amparo à Pesquisa do Estado de São Paulo, 2021/13050‐2; Conselho Nacional de Desenvolvimento Científico e Tecnológico, 306640/2023‐6, 134706/2024‐2; PROPe/Unesp, 13788.

## Conflicts of Interest

The authors declare no conflicts of interest.

## Data Availability

The data that support the findings of this study are available from the corresponding author upon reasonable request.
